# Commentary: Emotion Perception in Members of Norwegian Mensa

**DOI:** 10.3389/fpsyg.2019.01164

**Published:** 2019-05-22

**Authors:** Anja Vaskinn

**Affiliations:** ^1^Norwegian Centre for Mental Disorders Research, Oslo University Hospital, Oslo, Norway; ^2^Institute of Clinical Medicine, University of Oslo, Oslo, Norway

**Keywords:** social cognition, emotion perception, high intelligence, validity, assessment

Recently, an interesting paper by Egeland (2019) examined social cognition, indexed by a point-light display measure of emotion perception, in members of Mensa. It was concluded that Mensa members had better social cognition, in line with the defining feature of their group membership status, i.e., superior IQ, compared to a healthy community sample.

The social cognitive test in question, the Emotional Biological Motion (EmoBio) test (Heberlein et al., 2004) was standardized in Norwegian in an introductory paper some years ago (Vaskinn et al., 2016). The test uses a proportional scoring method (Couture et al., 2010) where a response is credited based on the proportion of a healthy reference sample that gives that response. When adopting the measure into Norwegian, it was deemed necessary to provide a Norwegian standardization instead of relying on normative data from other cultural contexts, such as the United States (Kern et al., 2013). Therefore, the distribution of scores in a Norwegian healthy reference sample (*n* = 101) was used to decide how a particular response should be scored (Vaskinn et al., 2016). Subsequent Norwegian publications on clinical populations (Engelstad et al., 2017, 2018; Vaskinn et al., 2017, 2018; Egeland et al., 2019) and the Mensa sample (Egeland, 2019) have used an algorithm based on this distribution to arrive at EmoBio scores. In the introductory paper, another group of healthy control participants (*n* = 84) was used in case-control comparisons with a group of individuals with schizophrenia (see Vaskinn et al., 2016 for details).

While the Egeland (2019) paper covers an important and fascinating topic, some issues need clarification. As an argument for the validity of the study findings, it is stated that the EmoBio scores of the healthy control sample, consisting of senior high school students and employees at a government agency, were similar to that of another Norwegian healthy control sample (Vaskinn et al., 2016). I assume the latter refers to the 84 healthy control participants described above. However, given that the difference between these two control samples amounts to a medium effect size (Cohen's *d* = 0.53), with the Egeland (2019) participants performing worse (Egeland mean total EmoBio score = 0.82, s.d. = 0.11; Vaskinn mean = 0.87, s.d. = 0.08), it is not immediately clear that this is the case. In addition, the EmoBio performance of the Mensa sample (mean = 0.86, s.d. = 0.08) is slightly below the Vaskinn et al. (2016) healthy control participants (Cohen's *d* = 0.13). This begs the question of whether the intact or superior social cognition of these Mensa members is an artifact of the comparison sample used. The answer has implications for how to answer to the overall question of the Egeland (2019) paper; does intellectual giftedness extend to social cognition, or is there a social cost to high intelligence?

Preferably, further arguments for the validity of the findings should be provided, so that the reader can be more confident that the results indeed suggest that this Mensa population is without social cognitive decrements.

## Author Contributions

AV conceived the idea, wrote the manuscript, and agrees to be accountable for the work.

### Conflict of Interest Statement

The author declares that the research was conducted in the absence of any commercial or financial relationships that could be construed as a potential conflict of interest.
